# Predicting the storage time of green tea by myricetin based on surface-enhanced Raman spectroscopy

**DOI:** 10.1038/s41538-023-00206-1

**Published:** 2023-06-09

**Authors:** Mengxuan Xiao, Yingqi Chen, Fangling Zheng, Qi An, Mingji Xiao, Huiqiang Wang, Luqing Li, Qianying Dai

**Affiliations:** State Key Laboratory of Tea Plant Biology and Utilization, Anhui Agricultural University, Hefei, 230036 Anhui China

**Keywords:** Chemical modification, Bioinorganic chemistry

## Abstract

The quality of green tea changes rapidly due to the oxidation and degradation of polyphenols during storage. Herein, a simple and fast Surface-enhanced Raman spectroscopy (SERS) strategy was established to predict changes in green tea during storage. Raman spectra of green tea with different storage times (2020–2015) were acquired by SERS with silver nanoparticles. The PCA-SVM model was established based on SERS to quickly predict the storage time of green tea, and the accuracy of the prediction set was 97.22%. The Raman peak at 730 cm^−1^ caused by myricetin was identified as a characteristic peak, which increased with prolonged storage time and exhibited a linear positive correlation with myricetin concentration. Therefore, SERS provides a convenient method for identifying the concentration of myricetin in green tea, and myricetin can function as an indicator to predict the storage time of green tea.

## Introduction

Green tea is a popular beverage worldwide because of its fresh flavour and biological activity. However, green tea ages and deteriorates over time, fresh flavour becomes stale, and its bright colour browns after storage due to the oxidation and degradation of polyphenols^1^. For example, flavonol glycosides hydrolyse during the storage of green tea and remove glycosides to form glycogen, and myricetin could accelerate the oxidation of EGCG (epigallocatechin gallate) during storage time and cause tea infusion browning^2,3^. The deterioration of green tea quality during storage reduces its commercial and health value, however no specific standards and characteristic compounds are available for the deterioration of green tea quality. Therefore, it is important to evaluate the quality change and find the characteristic compounds of green tea during storage.

Traditional sensory evaluation and chemical analysis are the main methods used to evaluate the quality of tea during storage. Traditional sensory evaluation requires professional experts to discriminate the quality of fresh and aged tea, which is highly efficient but depends on humans and can thus be subjective^4^. Changes in green tea quality during storage can be investigated by chemical analysis through examining compounds by GC‒MS^5^, HPLC^6^, LC‒MS^7^ during tea storage. Although sensory evaluation and chemical detection can detect changes in tea quality during storage, they all require professional skills and are time-consuming^8^. Therefore, developing a rapid and convenient approach to monitor changes in chemical compound during green tea storage is essential. Compared with sensory evaluation and chemical detection, spectral analysis is a rapid and convenient method; spectral analysis can be used to identify the quality change in green tea during storage. Based on hyperspectral imaging and near infrared spectroscopy, Li^9^ and Wang^10^ established a discrimination model to evaluate the storage quality of green tea. However, they did not find indicator compounds related to the storage time of green tea.

Surface-enhanced Raman spectroscopy (SERS) is a nondestructive method that requires minimal sample preparation and exhibits analytical advantages, such as rapid, objective and low detection limits^11^. Huang^12^ developed a linear, rapid and effective method for measuring catechin concentration by SERS and the intensity of the main Raman peak at 1328 cm^−1^ and found that catechin can be detected at µM levels by SERS with citrate-capped AgNPs. Qi^13^ analysed the quality changes in wine at different storage times by SERS and found that ethyl carbamate formed in the process of fermentation and storage of alcoholic beverages can be used as an important indicator, which could predict whether wine has deteriorated or not. Therefore, SERS is worth using to identify the change in green tea quality during storage.

In this study, the sensory quality and chemical compounds in green tea were investigated at different storage times, the Raman signal of green tea infusion was obtained by SERS with silver nanoparticles, an effective model to accurately predict the storage time of green tea was screened out, and the characteristic Raman peaks and indicator compounds were identified to predict green tea storage time by qualitive and quantitative analysis.

## Results

### Sensory evaluation and chemical analysis

The sensory characteristics of TPHK samples with different storage times based on the hedonic 9-point scale are shown in Supplementary Table 1. There were significant differences between tea samples (*p* < 0.05), but the 2017 and 2018 tea samples showed no significant difference in aroma and taste. The scores for all four sensory factors decreased as the storage time increased, and the overall acceptability of tea samples decreased sharply in 2015. Although the origin of the tea leaves was consistent, the compounds still slowly oxidised and hydrolysed under storage conditions at 4 °C^4^, resulting in differences in the quality of TPHK with different storage times.

The compound contents of TPHK samples with different storage times were detected and analysed, and the results are shown in Supplementary Table 2. The most abundant compound in the tea sample was EGCG, and the least abundant compound was myricetin. With increasing storage time, the contents of GA, GC, EGC, EGCG, GCG and ECG decreased, the contents of C and M increased, and the CAF content remained unchanged. The compound contents of raw Pu-erh teas also obtained similar results during storage^14^. Polyphenols oxidised during storage reduce the freshness of green tea^10^.

**Fig. 1:**
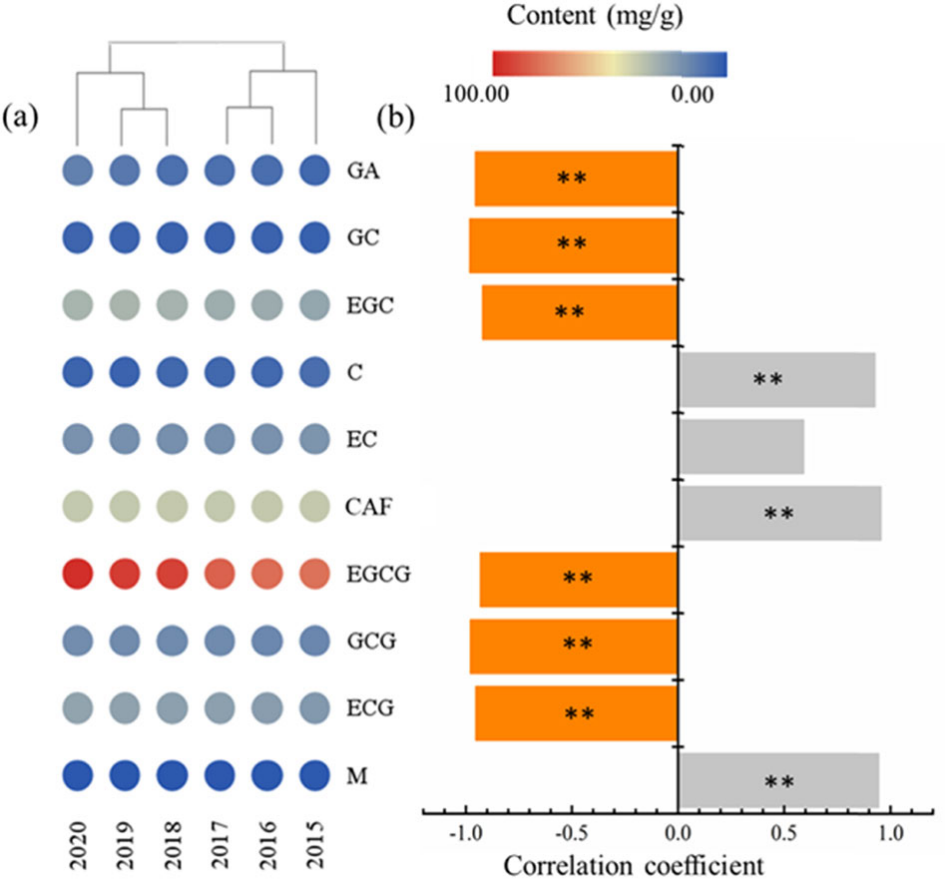
Sensory evaluation and chemical analysis. HCA heatmap of TPHK compounds at different storage times (**a**) and correlation analysis of compound content and overall acceptability (**b**), *p* < 0.01.

Polyphenols in TPHK samples were analysed by hierarchical cluster analysis (HCA). The results are shown in Fig. 1a. Tea samples were divided into two groups overall, including tea samples from 2020 to 2018 and 2017 to 2015. Among them, the tea samples in 2020 were different from those in 2019 and 2018, and the tea samples in 2015 were different from those in 2017 and 2016. The overall acceptability and content of TPHK samples were analysed by Pearson correlation analysis, and the results are shown in Fig. 1b. Polyphenol contents were closely related to the overall acceptability score of tea. The overall acceptability of tea taste was negatively correlated with the contents of GA, GC, EGC, EGCG, GCG and ECG, and the coefficient was above 0.95 (*p* < 0.01). There was a significant positive correlation between taste and C, CAF and M, and the coefficient was above 0.95 (*p* < 0.01). However, sensory evaluation or chemical analysis can only show that there were differences in the quality of TPHK at different storage times.

### SERS analysis

Supplementary Fig. 1 presents the SEM and EDS results of the prepared silver nanoparticles. As depicted in Supplementary Fig. 1A, the EDS elemental analysis of the AgNPs revealed that the prepared SERS substrates principally contained AgNPs with high purity. As shown in Supplementary Fig. 1B, the obtained AgNPs were mainly distributed in the range of 36–52 nm, and the average diameter was ~43.3 nm. The size, shape, and aggregation pattern of AgNPs affect the enhancement of the Raman signals of target analytes. Lok et al. ^15^ found that compared with large silver nanoparticles, small silver nanoparticles not only exhibit stronger antibacterial properties but also show better stability. Therefore, silver nanoparticles can be used to enhance the Raman signal of tea infusion.

**Fig. 2:**
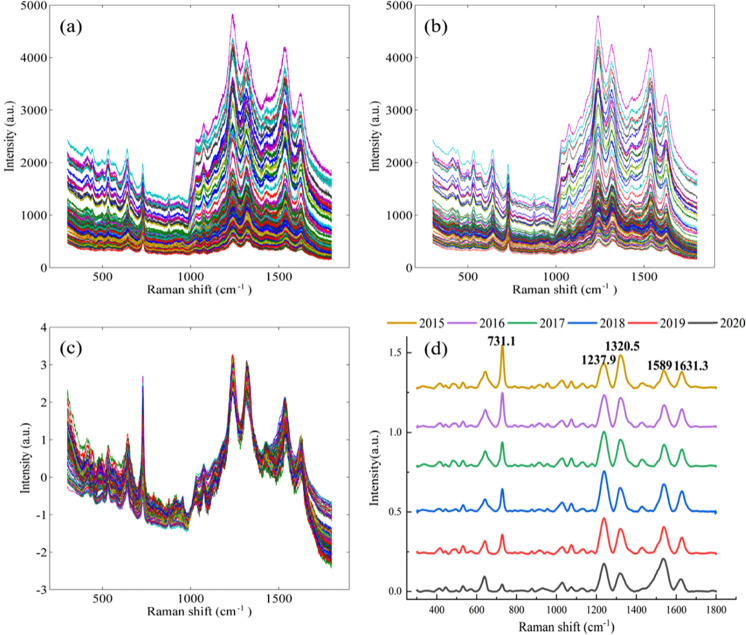
Spectral pretreatment. Raman spectra of TPHK obtained using raw data (**a**), S-G smoothing preprocessed data (**b**), S-G smoothing and SNV preprocessed data (**c**) and averaged data over 6 years (**d**).

Preprocessing the spectral data is necessary and will reduce noise and fluorescence interference in the process of spectral acquisition. Figure 2a shows a raw Raman spectra with noise. S-G smoothing was applied with minor noise interference elimination (Fig. 2b). The SNV method was further applied, and the results are shown in Fig. 2c. It significantly eliminates noise interference and smooths the whole spectrum. The Raman spectra after preprocessing were used for the next analysis and modeling.

The average Raman spectra of the TPHK samples are shown in Fig. 2d. In line with the storage time, the Raman spectra generally maintained their basic structures, but all major Raman spectra gradually changed. The intensities of the Raman peaks at 731.1 cm^−1^ and 1320.5 cm^−1^ increased, while the Raman peaks at 1237.9, 1589 and 1631.3 cm^−1^ decreased. Attributions of the major Raman peaks are shown in Supplementary Table 3. There are some differences in Raman shifts between this paper and others. This may be the changes in the structure or content of the silver nanoparticle material, resulting in “blueshift” and “redshift” phenomena^12^.

The vibrations at 731.1, 1589 and 1631.3 cm^−1^ were caused by polyphenols^11,12^. Catechins are the most abundant, accounting for 60–80% of the total tea polyphenols, and the second most abundant are flavonoids. Catechins are flavanols, a class of 2-phenylbenzopyran derivatives. During the storage of green tea, the phenolic hydroxyl group in the catechin B-ring is oxidised to O-quinone^13^. O-quinone is further polymerised to form a compound with a benzophenone structure. Moreover, C^5^-OH and C^7^-OH groups in the A-ring of catechin are prone to polymerise and form dimers of di-flavanol^13^. Therefore, when the storage time of green tea increased, the intensity of the main Raman peak at 730 cm^−1^ increased due to the stretching vibrations of polyphenol C-H moieties and benzene rings. The intensity of the main Raman peaks at 1589 and 1631.3 cm^−1^ decreased due to the stretching vibration of the benzene ring.

The Raman peak at 1237.9 cm^−1^ was caused by the stretching vibration of a hydrocarbon double bond ( = C-H)^11^. During the storage of green tea, the unsaturated bonds in unsaturated fatty acids could be oxidised into saturated bonds under the influence of the external environment^12^, which leads to a decrease in the strength of the Raman peak at 1237.9 cm^−1^. The peak at ~1320.5 cm^−1^ was caused by the bending vibrations of carbohydrate CH_2_ groups^15^, and with increasing storage time, the Raman peak at 1320.5 cm^−1^ may be produced from the degradation of polysaccharides in tea to soluble sugars^16^.

The above results show that SERS can detect the Raman spectra of TPHK tea infusions, and the Raman spectra of TPHK were different with different storage times, which can be used to identify the quality of TPHK during storage by establishing an effective discrimination model.

### Model analysis and comparison

**Fig. 3:**
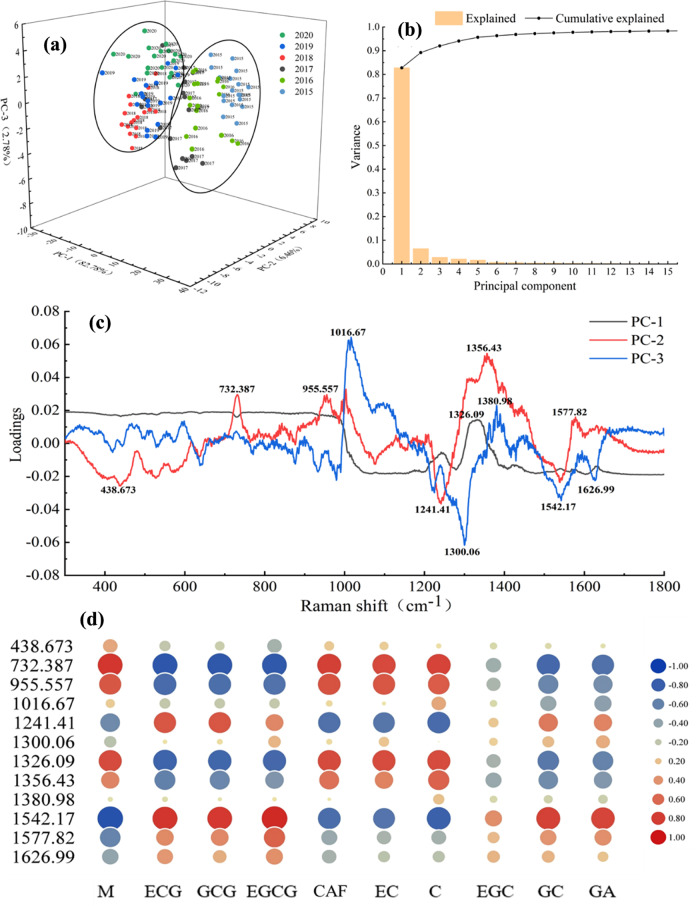
Correlation analysis of SERS and polyphenols. Principal component analysis score plot of all spectral data (**a**), explained variance and cumulative explained variance of spectral data by PCA (**b**), loading plot (**c**), and heatmap of correlation between the major Raman peaks and compound contents (**d**).

In this paper, PCA was used to analyse the Raman spectra of TPHK samples range from 2020 to 2015, and the results are shown in Fig. 3. Figure 3a shows PCA score cluster plots for TPHK samples from different storage times based on SERS. The first three principal components accounted for 92.02% of the variance (Fig. 3b). The total contribution rate represents the primary spectral information. According to the first principal components, all samples were divided into two cluster areas. The 2020–2018 samples constitute a cluster area, and the 2017–2015 samples constitute another cluster area.

To more accurately distinguish the storage time of TPHK, PCA-LDA and PCA-SVM models were established by the full spectral data. Before modeling, the Kennard–Stone method was used to divide the samples of each group into a calibration set (72 samples) and a prediction set (36 samples) with a ratio of 2:1. PCA-LDA and PCA-SVM models were established, and the results are shown in Table 1. The accuracy of the calibration and prediction set of the PCA-LDA model was lower than that of PCA-SVM (Table 1). This may be because LDA is the linear separation approach. This means that a straight line (a vector) separates two classes, and samples may be falsely assigned to the wrong class^17^. In this context, SVM is a nonlinear classification algorithm. From the sensory evaluation results, we can see that the quality differences among samples were very small, so the nonlinear SVM method was more conducive to distinguishing the quality of TPHK at different storage times. Therefore, when the number of PCs was 11, the accuracy of the PCA-SVM (c = 111.4305, g = 0.0010) model was higher, the accuracy of the calibration set was 98.61%, and the accuracy of the prediction set was 97.22%.

**Table 1:** Model comparison of TPHK in different storage time based on SERS.

Spectral pre-process	Model	PCs	Parameter		Calibration set		Prediction set	
			c	g	Result^a^	Accuracy	Result	Accuracy
SG + SNV	PCA-LDA	4	\	\	58/72	80.56%	25/36	69.44%
	PCA-SVM	6	111.4305	0.001	71/72	98.61%	35/36	97.22%

^a^m/n means m samples were correctly classified in a total number of n samples.

### Correlation analysis of SERS and polyphenols

Raman spectra are produced by the vibration of chemical bonds in compounds. Therefore, it is necessary to explore the correlation between compounds and Raman spectra. The loading plot of the first three principal components for the Raman spectra showed that 12 main Raman peaks distinguished the storage time of TPHK, which were 438.673, 732.387, 955.557, 1016.67, 1241.41, 1300.06, 1326.09, 1356.43, 1380.98, 1542.17, 1577.82 and 1626.99 cm^−1^ (Fig. 3c). The results obtained from correlation analysis of the 12 major Raman peaks and the contents of compounds are shown in Fig. 3d. The larger the circle is, the greater the absolute value of the correlation coefficient. Red indicates a positive correlation, and blue indicates a negative correlation. The correlation laws for the four Raman peaks at 732.387, 955.557, 1326.09, and 1356.43 cm^−1^ were consistent. They were positively correlated with M, EC and C but negatively correlated with ECG, GCG and EGCG. The Raman peaks with high correlations were those at 732.387 cm^−1^ and 1326.09 cm^−1^. The three major Raman peaks at 1241.4, 1542.17, and 1577.82 cm^−1^ were positively correlated with ECG, GCG and EGCG contents but negatively correlated with CAF, EC and C. The Raman peak with the highest correlation was that at 1542.17 cm^−1^. The five Raman peaks at 438.673, 1016.67, 1300.06, 1380.98 and 1626.99 cm^−1^ exhibited little correlation. These Raman peaks were possibly caused by vibrations of other substances, but further study is needed.

### Identifying the storage time of TPHK based on key compounds by SERS

Among the major Raman peaks mentioned above, 732.387, 1326.09 and 1542.17 cm^−1^ were highly correlated with the contents of compounds. It was shown that 732 cm^−1^ and 1576 cm^−1^ were caused by the C-H and benzene-ring vibrations of polyphenols, and 1326 cm^−1^ was caused by the CH_2_ vibration of sugars^12,13^. The Raman spectra of the most popular tea polyphenols, such as EGCG, ECG, GCG, EGC, kaempferol- and myricetin, are shown in Supplementary Fig. 2. The peaks at 1326.09 and 1542.17 cm^−1^ were all detected in polyphenols, while a major Raman peak at 730 cm^−1^ was detected only in myricetin, which was a characteristic peak. Therefore, myricetin may be a characteristic compound to predict the storage time of TPHK. This result is consistent with our previous study, and myricetin content increased in green tea infusion by HPLC after long-term thermal treatment^3^. Therefore, with the extension of storage time, the content of myricetin in green tea will increase, and the Raman peak intensity at 730 cm^−1^ will increase.

**Fig. 4:**
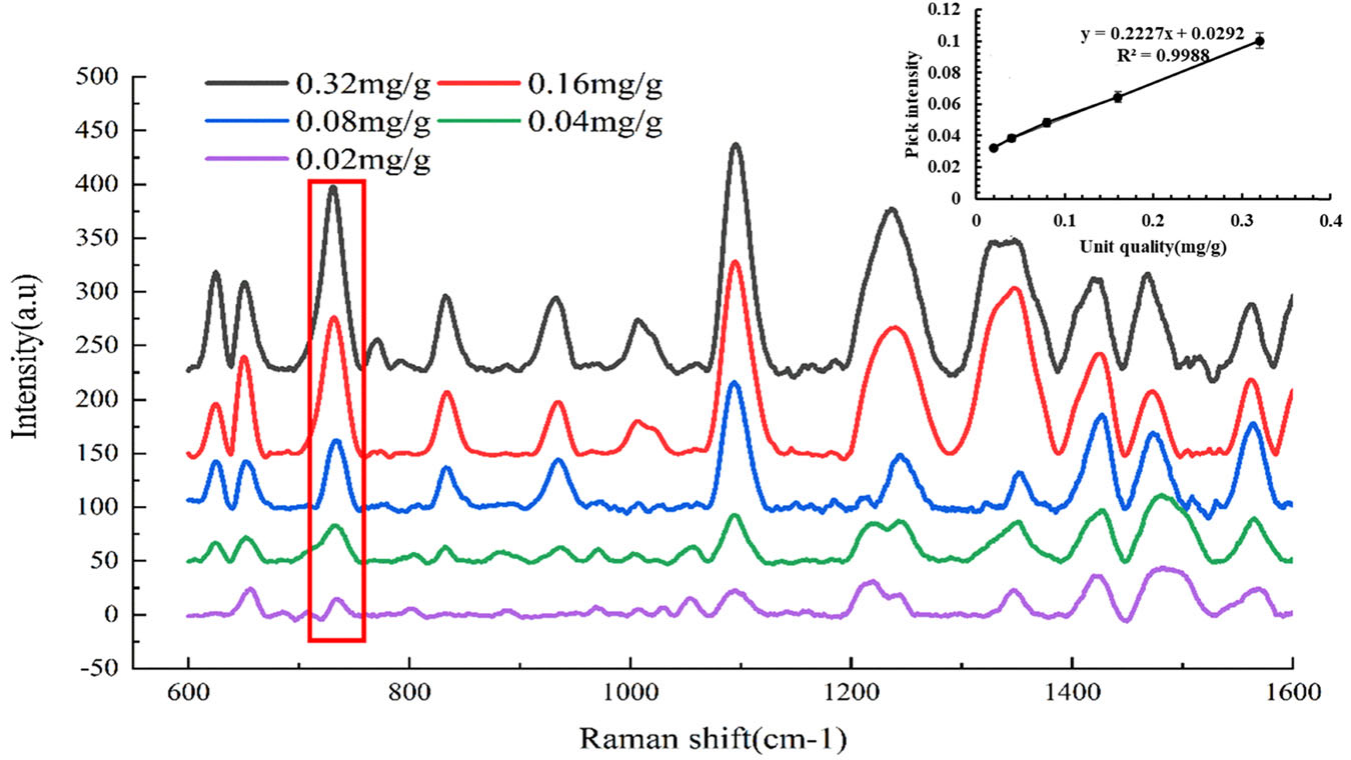
Standard curve of myricetin. Raman spectra and standard curves of myricetin at different concentrations.

In addition, we measured the Raman intensity of myricetin at 730 cm^−^^1^ and established the correlation curve. Myricetin standards at different concentrations were dropped onto gold-coated glass slides and subjected to Raman spectroscopy (Fig. 4). A standard curve was generated using the known myricetin concentration as the abscissa and the peak intensity (730 cm^−1^ peak) of the myricetin as the ordinate (Fig. 4; inset). The 730 cm^−1^ intensity is linearly positively correlated with the concentration (0.02–0.32 mg/g) of myricetin. When the concentration of myricetin was 0.02 mg/g, some Raman peaks almost disappeared. There was a good linear positive correlation between the Raman intensity and the concentration of myricetin. The concentration of myricetin was calculated by the Equation y = 0.2227x + 0.0292, with a correlation coefficient of *R*^2^ = 0.9988.

**Fig. 5:**
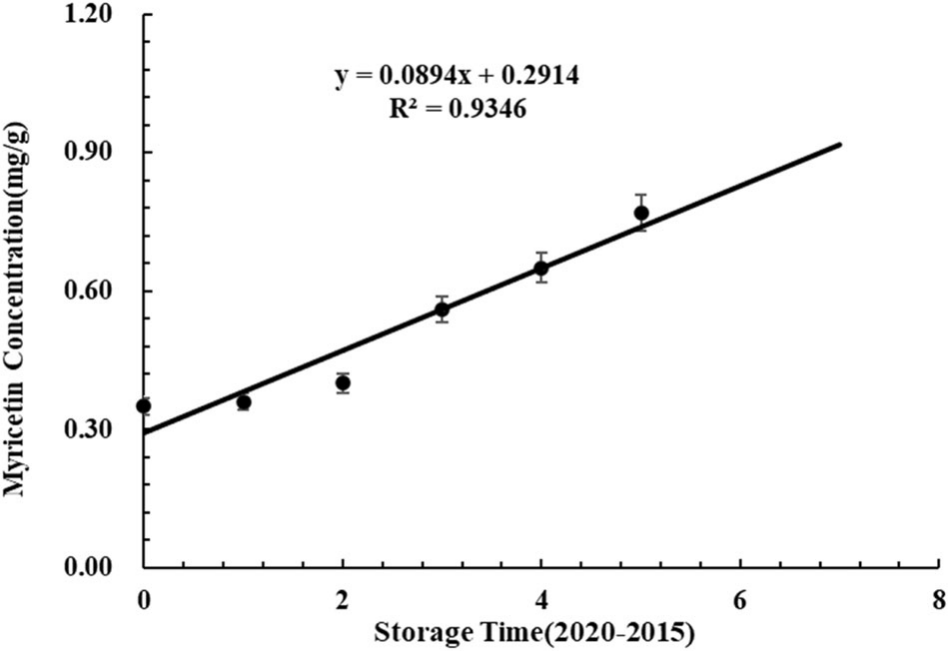
Linear correlation analysis of myricetin and storage time. Linear correlation curves between myricetin concentration and storage time.

The myricetin concentration of each tea sample was calculated and is listed in Table 2. The myricetin content was also detected by SERS and UPLC. There were significant differences in the content of myricetin in TPHK from 2015 to 2020, as detected by UPLC. The concentrations of myricetin detected by the two methods were similar, with a maximum error of 5.81%. SERS provides a simple and convenient method to detect the concentration of myricetin in green tea. To further verify the relationship between myricetin and the storage time of green tea, linear correlation analysis was carried out between the concentration of myricetin and the storage time of green tea. The concentration of myricetin exhibited a good linear positive correlation with storage time, with *R*^2^ = 0.9346 (Fig. 5). Therefore, myricetin is the characteristic compound of the quality change of green tea during storage and can be used as an indicator to predict the storage time of green tea.

**Table 2:** Quantitative analysis of myricetin in TPHK by SERS and UPLC.

Years	SERS (mg/g)	UPLC (mg/g)	Average error
2020	0.35 ± 0.04	0.35 ± 0.00^a^	0.40%
2019	0.36 ± 0.06	0.40 ± 0.00^b^	3.73%
2018	0.40 ± 0.02	0.45 ± 0.06^c^	4.59%
2017	0.56 ± 0.01	0.60 ± 0.02^d^	3.78%
2016	0.65 ± 0.01	0.70 ± 0.00^e^	5.04%
2015	0.77 ± 0.04	0.71 ± 0.02^f^	5.81%

Different letters represent significant differences (*p* < 0.05).

## Discussion

Green tea is easily oxidised and degraded during storage and transportation, so its taste and aroma appear stale, thus reducing its commercial value. Many methods are used to measure the quality of green tea during storage. Zhou^14^ detected the change in green tea quality during storage by LC‒MS and found that the total amount of polyphenols showed a downwards trend, while myricetin showed an upwards trend. This method was used to analyse the change in compounds in green tea during storage but did not identify the compounds related to storage. Dai^18^ detected the change in volatile compounds in stale green tea by GC‒MS. It was found that the aroma stale odour of green tea was responsible for 1-octen-3-ol, benzyl alcohol, benzaldehyde, etc., but there was no relationship with the storage time of green tea. These methods involve a long experimental time and a complicated data analysis process. To solve this problem, most of the studies on the quality of green tea during storage are based on models^9,10^. Information on the near infrared spectrum^19^ and hyperspectral spectrum^20^ of green tea can be used to identify the storage time of green tea. However, this method is based on spectral information and has not identified the indicative compounds.

In our study, we detected the quality of green tea at different storage times by SERS and found that the Raman peak at 730 cm^−1^ showed a good linear positive correlation with the green tea storage time. The Raman peak at 730 cm^−1^ is caused by the vibration of C-H in polyphenols^17,21^, and compared with other polyphenols, the Raman peak at 730 cm^−1^ only appears in myricetin. Myricitrin will be hydrolysed during the storage of green tea^14^, and the glycoside will be removed to form myricetin, which causes an increase in myricetin content in green tea during storage (Supplementary Fig. 2). Myricetin and EGCG exhibited synergistic effects to redden green tea samples^3^, which led to stale green tea. Therefore, myricetin is an important compound found during the change in green tea quality during storage.

To further verify whether 730 cm^−1^ is caused by the myricetin content in green tea during different storage times, we also used the external standard curve of myricetin established by SERS to compare the myricetin content in tea infusion with the UPLC and found that the error was ~5%. Therefore, the Raman peak at 730 cm^−1^ caused by myricetin can be used as an indicator for the change in green tea quality during storage. This method can be used to predict the storage time of green tea quickly and conveniently.

Although this study has established a simpler and more rapid method to identify the quality of green tea during storage based on SERS, there are still limitations that must be resolved. Therefore, we suggest that in future research, (1) The accuracy of this model should be verified and improved by judging more green tea with different storage times. (2) A more convenient and instant method should be developed for determining the quality of green tea during storage based on portable Raman technology.

In this paper, a method was first developed to acquire Raman spectra of green tea by SERS with silver nanoparticles. The SERS combined with PCA-SVM model can quickly and accurately predict the storage time of green tea more than sensory evaluation and chemical analysis, and the accuracy of the prediction set is 97.22%. The Raman peak at 730 cm^−1^ was a characteristic peak of myricetin, and the intensity of 730 cm^−1^ had a linear positive correlation with myricetin concentration during green tea storage. In addition, the concentration of myricetin also showed a good linear positive correlation with the green tea storage time, so myricetin could be the characteristic compound for the change in green tea quality during storage. Therefore, this work provides a quick and accurate PCA-SVM model to predict the storage time by SERS data; in addition, it was determined that the Raman peak at 730 cm^−1^ caused by myricetin can be used as an indicator to predict the storage time of green tea.

## Methods

### Tea samples and chemicals

TPHK is an typical and high economic efficiency green tea in China. We collected green tea samples (TPHK, Anhui) from 2015 to 2020 with the same tea variety and grade tea fresh leaves. Tea samples were stored in a 4 °C refrigerator.

( + )-Catechin (C), (−)-epicatechin (EC), (−)-epigallocatechin (EGC), (−)-gallocatechin (GC), (−)-epicatechin gallate (ECG), (−)-gallocatechin gallate (GCG), (−)-epigallocatechin gallate (EGCG), GA, caffeine, quercetin, kaempferol and myricetin (M) were purchased from Sigma‒Aldrich (St. Louis, MO, USA). HPLC-grade methanol and acetonitrile were purchased from Tedia Co., Ltd. (Fairfield, OH, USA).

### Sensory evaluation

Sensory evaluation was carried out according to the Chinese national standard procedure for evaluating tea leaves (GB/T 23776, 2018)^22^. Briefly, a 3.0 g sample was infused in 150 mL of boiled water for 4 min. The tea infusion was then transferred to a porcelain cup placed in an insulated container for sensory evaluation. The professional sensory panel consisted of 6 members (one man and five women). All sensory panels had more than 6 years of experience in tea sensory evaluation. Quality attributes consisted of tea infusion colour, aroma, taste, and overall acceptability. Sensory panels all did not know the sample information, and the samples were presented randomly. Tap water was provided for each panel member to clean their palate between evaluations for each sample. The hedonic 9-point scale was used to rate samples (9 = like extremely; 8 = like very much; 7 = like; 6 = like slightly; 5 = neutral; 4 = dislike slightly; 3 = dislike moderately; 2 = dislike; and 1 = dislike extremely)^23^.

### Sensory tests

The project was submitted and approved by the Research Ethics Committee of the University of Anhui Agricultural University. All subjects signed a consent form to participate in the sensory and consumer tests.

### Chemical analysis

Extraction of chemical compounds from tea leaves was carried out according to the China National Institute of Standardization standard GB/T 8304-2013^24^. The amounts of caffeine, GA, and catechins, including C, EC, EGC, GC, ECG, GCG and EGCG, as well as myricetin, were determined by UPLC with a Waters H-Class 2489 series. The UPLC system (Waters Corporation, Milford, MA, USA) consisted of a Phenomenex C_18_ guard column (2.1 × 100 mm, 1.8 μm; Phenomenex, Torrance, CA, USA) protected with an Agilent C_18_ chromatography column (2.1 × 5 mm, 1.8 μm), and analyses were performed according to the method described by Nian^25^.

### SERS data acquisition and processing

The ratio of tea to water was 1:16, the tea was soaked in a 70 °C water bath for 30 min, and the tea leaves were filtered. Silver nanoparticles were prepared according to the method of Liu^26^. First, 2 mL 2.0 g/L AgNO_3_ solution and 1 mL 10 g/L Na_3_C_6_H_5_O_7_·2H_2_O solution was dissolved in 50 ml water. Then, the mixture was heated in a microwave oven for 10 min, removed, and cooled for further experiments. The tea infusion and silver nanoparticles were mixed in a 1:1 ratio and then analysed by SERS^27^^.^

To test the morphology of the prepared silver nanoparticles, 2 μL of silver nanoparticle solution was first dropped onto 5*5 mm silicon wafers, dried and observed by scanning electron microscopy (SEM) (JSM-7800F, JEOL Corporation, Japan) to determine their shape and particle size. The size of the silver nanoparticles was calculated by measuring the length and width of AgNPs from SEM images using particle size distribution software. (Nano Measurer 1.2.5, Department of Chemistry, Fudan University, Shanghai, China). Elemental analysis of AgNPs was performed by energy dispersive spectroscopy (EDS, Hitachi, Japan)^28^.

A Horiba Jobin Yvon Lab RAM HR Evolution Raman spectrometer was used. A silicon wafer with a Raman band at 520.7 cm^−1^ was used to calibrate the spectrometer. The sample to be tested was dripped onto a gold-coated glass slide^28^. The settings of the microspectrometer were 10× microscope objective, 200 μm slit width, 600 lines/mm, and 2 s integration time. In this experiment, the total integration time was 6 s. Each spectrum was collected in the spectral range 300–1800 cm^−1^ through LabSpec6 Software (Horiba). A total of 108 spectral data points were collected. To prevent fluorescence interference, S-G smoothing and SNV were used for spectral pretreatment.

### Establishing the discriminant model

Principal component analysis (PCA) can reduce the principal component dimension of complex multivariable data to a great extent, transform the complex multidimensional spatial data into a visual distribution scatter diagram, objectively capture the minimum spectral differences between similar spectra, distinguish the relationship between different spectra, and analyse and compare their similarities^29^. On this basis, the scores plot and loadings plot are generated using principal components (PCs), which have been used to explain the variability in those original datasets.

Linear discriminant analysis (LDA) classifies the dependent variable by dividing an n-dimensional feature space into two regions that are separated by a hyperplane, which is denoted by a linear discriminate function^30^. In LDA, observations from each case are compared with others to provide models for grouping data. LDA is a very powerful supervised learning technique through which data is efficiently sorted into classes based on the distances between them. LDA is mostly used when no priori hypotheses exist regarding the data and during initial exploratory phases of research^31^. In a sense, LDA analysis suggests the most significant solution possible. The shorter the distance between the different classes, the more similarities the samples exhibit. The result of the discriminant model is expressed as the correct classification rate, which varies between 0% and 100%, with larger values indicating superior model performance.

The support vector machine (SVM) algorithm is an effective and widely applied supervised classification method. The SVM algorithm is focused on obtaining the “optimal” boundary of two classes in a vector space independent of the probabilistic distributions of training vectors in the data set^10^. A commonly employed kernel function is the radial basis function, for which the penalty factor c and kernel parameter g must be optimised. In this study, both parameters were set within 2^−10^–2^10^ and optimised through a grid search procedure, and fivefold cross-validation was used to prevent overfitting^32^. The result of the discriminant model is expressed as the correct classification rate, which is between 0% and 100%, with larger values indicating superior model performance^33^.

### Statistical analysis

Data are expressed as the mean ± standard deviation of triplicate tests. Sensory evaluation data were subjected to analysis of variance (ANOVA) followed by Duncan’s test. Line plots and Raman spectra were drafted using Origin 9.0 (Origin Lab Corp., Northampton, MA, USA). Data acquisition and processing were achieved using LabSpec6 software™ (Horiba Jobin Yvon), and a heatmap was generated using TBtools software (https://github.com/CJ-Chen/TBtools). All multivariate data analyses, including PCA, LDA and SVM modeling, were performed using MATLAB R2014a (The MathWorks, Natick, MA, USA).

## Supplementary information

Supplementary materials

nr-reporting-summary

## Data Availability

All data generated and analysed during this study are included in this published paper.
