# Supplementary material for: Predicting the storage time of green tea by myricetin based on surface-enhanced Raman spectroscopy
Source: NPJ Sci Food. 2023 Jun 9;7:28. doi: 10.1038/s41538-023-00206-1 (PMC10250423; doi:10.1038/s41538-023-00206-1)
Supplement: Supplementary file 1 — Supplementary materials [file 41538_2023_206_MOESM1_ESM.pdf]

Supplementary materials Captions

Supplementary Table 1 Sensory evaluation of TPHK green tea at different storage times.

Supplementary Table 2 Compound content of TPHK at different storage times.

Supplementary Table 3 Raman shift (cm-1) and assignments of the main Raman bands of TPHK.

Supplementary Figure 1 SEM of AgNPs. (A) EDS elemental analysis diagram of silver nanostructures. (B) SEM images of different sizes of silver nanoparticles (1 μm and 500 nm). (B1) Histogram of AgNP size distribution.

Supplementary Figure 2 Raman spectra of tea polyphenols.

Supplementary Figure 3 Hydrolysis and depigmentation of myricitrin.

Supplementary Table 1 Sensory evaluation of TPHK green tea at different storage times

Years	Color	Aroma	Taste	Overall acceptability
2020	9.00±0.00[f]	8.83±0.08[e]	9.00±0.00[e]	9.51±0.17[f]
2019	8.00±0.00[e]	7.67±0.17[d]	7.67±0.17[d]	8.52±0.26[e]
2018	6.33±0.17[d]	6.17±0.08[c]	6.17±0.08[c]	7.83±0.39[d]
2017	5.17 ± 0.08[b]	6.00 ± 0.00[c]	6.00 ± 0.00[c]	6.83 ± 0.01[c]
2016	5.67 ± 0.17[c]	5.00 ± 0.00[b]	5.17 ± 0.08[b]	6.03 ± 0.43[b]
2015	3.00 ± 0.00[a]	4.00 ± 0.00[a]	3.83 ± 0.08[a]	3.50 ± 0.25[a]

Figures with different letters within the same row differ significantly ($p<0.05$).

Supplementary Table 2 Compound content of TPHK at different storage times

Compounds (mg/g) [a]	2020	2019	2018	2017	2016	2015
C	4.34±0.02	4.05±0.02	5.47±0.01	5.42±0.01	5.72±0.01	7.35±0.02
M	0.35±0.04	0.40±0.01	0.45±0.04	0.60±0.02	0.70±0.01	0.71±0.04
EC	17.86±0.04	17.28±0.01	17.33±0.02	17.20±0.04	17.91±0.01	18.81±0.04
GA	12.79±0.01	10.53±0.01	7.84±0.02	7.86±0.04	7.43±0.01	5.45±0.01
GC	4.51±0.01	4.21±0.04	3.96±0.02	3.89±0.01	3.74±0.01	3.46±0.04
GAF	34.34±0.01	34.34±0.01	34.38±0.01	34.37±0.01	34.41±0.02	34.46±0.03
EGC	28.30±0.01	28.56±0.04	27.74±0.01	26.07±0.02	25.59±0.04	23.83±0.01
ECG	23.19±0.02	22.79±0.01	22.26±0.02	22.06±0.01	21.34±0.04	19.92±0.01
GCG	16.31±0.01	16.22±0.01	15.79±0.01	15.71±0.02	15.07±0.01	14.58±0.02
EGCG	88.10±0.01	84.66±0.01	83.37±0.01	75.93±0.02	73.35±0.04	72.25±0.02

[a] Data are mean ± standard deviation (n = 3)

Supplementary Table 3 Raman shift (cm^{-1}) and assignments of the main Raman bands of TPHK

Compounds	Observed Raman shift/cm^{-1}	Assignment	Reference
Polyphenols	732.1	γ (CH_2), γ (b- ring)	Huang et al., 2018<br>Guo et al., 2022<br>Qi et al., 2018
	959.2, 1013.1, 1016.1, 1044.3	γ (C-H), γ (b- ring)	
	1493.1, 1463.0, 1495.0, 1480.0	γ (b- ring), υ (OH)	
	1606.1, 1576.101, 1607.1	υ (b- ring)	
Sugars	1335.0, 1328.1, 1333.0	δ (CH_2)	
Unsaturated fatty acids	1254.1, 1249.11, 1253.12, 1200.0	υ (C-H)	

Assignments: υ, stretching; δ, skeleton in-plane bending; γ, skeleton out of plane bending.

Figure. S1

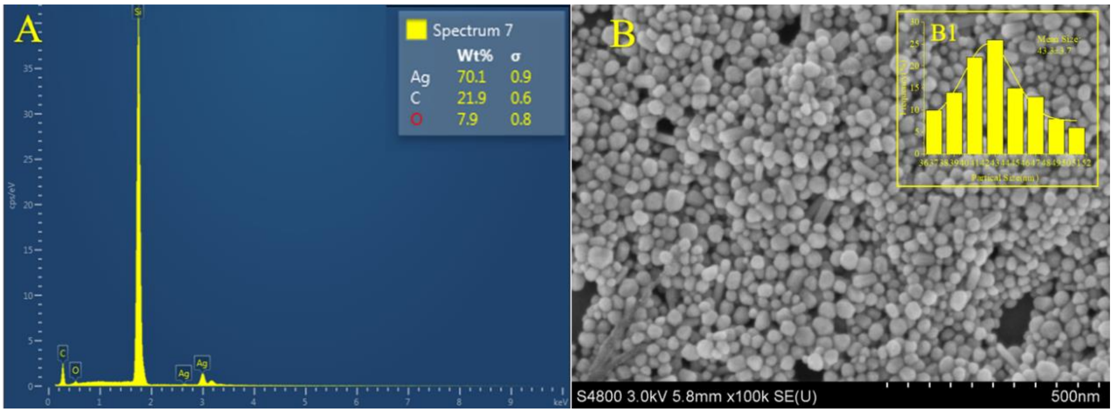

Supplementary Figure 1 SEM of AgNPs. (A) EDS elemental analysis diagram of silver nanostructures. (B) SEM images of different sizes of silver nanoparticles (1 μm and 500 nm). (B1) Histogram of AgNP size distribution.

Figure. S2

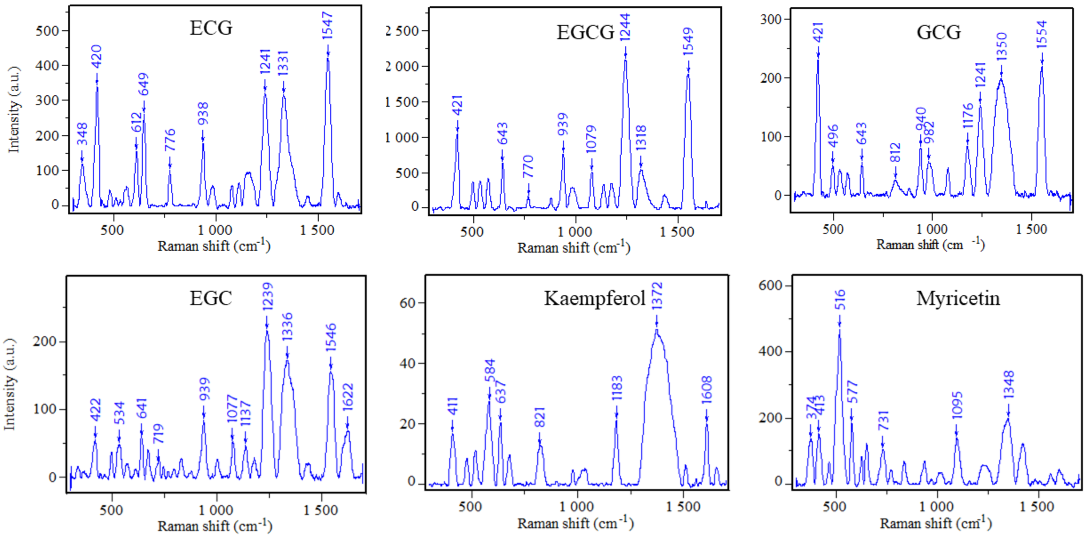

Supplementary Figure 2 Raman spectra of tea polyphenols.

Figure. S3

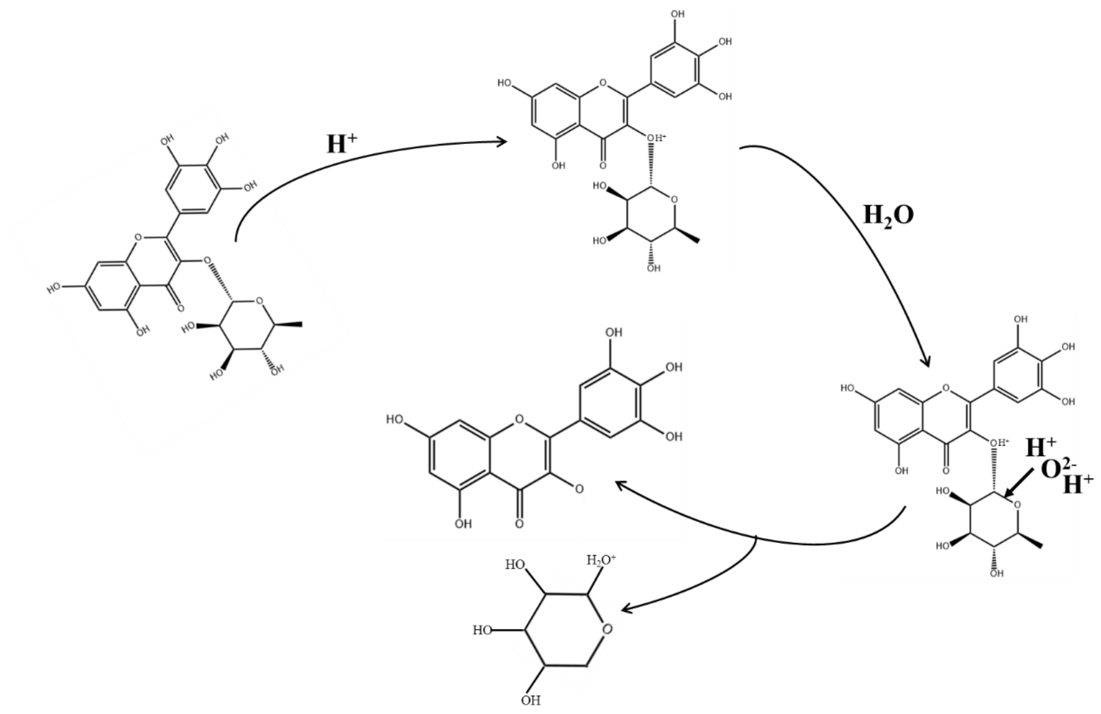

Supplementary Figure 3 Hydrolysis and depigmentation of myricitrin.
